# Cannabidiol protects lung against inflammation and apoptosis in a rat model of blunt chest trauma via Bax/Bcl-2/Cas-9 signaling pathway

**DOI:** 10.1007/s00068-025-02767-0

**Published:** 2025-02-07

**Authors:** Akın Süleyman Emre, Savran Mehtap, Doğan Cem, İlhan İlter, Arlıoğlu Melih, Özmen Özlem, Sezer Serdar, Çamaş Hasan Ekrem, Yazkan Rasih

**Affiliations:** 1 Department of Thoracic Surgery, Faculty of Medicine, Suleyman Demirel University, Isparta, Turkey; 2 Department of Medical Pharmacology, Faculty of Medicine, Suleyman Demirel University, Isparta, Turkey; 3 Department of Biochemistry, Faculty of Medicine, Suleyman Demirel University, Isparta, Turkey; 4 Department of Pathology, Faculty of Veterinary Medicine, Burdur Mehmet Akif Ersoy University, Burdur, Turkey; 5 Natural Products Application and Research Center (SUDUM), Suleyman Demirel University, Isparta, Turkey

**Keywords:** Cannabidiol, Chest trauma, Apoptosis, Mitochondrial stress, Lung, Contusion

## Abstract

**Purpose:**

This study aimed to investigate the hypothesis that cannabidiol (CBD), with known anti-inflammatory and anti-apoptotic effects, would reduce the severity of acute lung injury in pulmonary contusion following blunt chest trauma.

**Methods:**

Forty male Wistar Albino rats were randomly divided into four groups, each consisting of 10 rats: Sham, Trauma, Trauma + CBD, and CBD. The rats were treated with a single dose of 5 mg/kg CBD intraperitoneally 30 min before trauma. Then, the trauma were exposed to a weight of 200 g and a height of 1 m. After sacrifice, the lung tissues were removed for histopathological, immunohistochemical, biochemical, and genetic analyses.

**Results:**

Pulmonary injury of trauma group led to increases in tumor necrosis factor α, caspase-3, caspase-9, Bcl-2-associated X protein expressions, total oxidant status, oxidative stress index levels, and decreases in B-cell lymphoma expression and total antioxidant levels. Additionally, inflammatory cell infiltration, damage-related emphysema, pronounced hyperemia, and increased septal tissue thickness were observed histopathologically. CBD treatment ameliorated all these findings.

**Conclusion:**

CBD reduces lung damage in lung contusions caused by blunt chest trauma through its anti-inflammatory and antiapoptotic effects. More detailed studies investigating other important intracellular pathways are needed.

## Introduction

Trauma is a threat that can affect people in all age groups [1]. It is the most widespread cause of mortality in the first forty years of life and the third most common cause of death over forty [2]. Whereas thoracic trauma is the main cause of approximately %25 of these deaths, it is a contributing factor in the other %25 [1, 3].

Thoracic trauma damage can vary from a basic, isolated rib fracture to extensive vascular hemorrhage that necessitates a thoracotomy. The most common intrathoracic disease associated with blunt trauma is pulmonary contusion. Trauma-induced alveolar distension, alveolar rupture, separation of alveoli from bronchioles, intra-alveolar hemorrhage, and interstitial edema were named as pulmonary contusion. If the contusion is massive, major complications such as aspiration, bacterial pneumonia, and acute respiratory distress syndrome may develop [4–6].

Acute lung injury (ALI) secondary to pulmonary contusion is thought to occur in two ways; an increased inflammatory response and apoptosis. The inflammatory process, which is the first pathway, involves many different mechanisms of action including the innate inflammatory response, aggregation of leukocytes, activation of tissue macrophages, increased free oxygen radicals, arachidonic acid metabolites, and cytokines related to nuclear factor-kappa B (NF-κB) activation, and activation of proinflammatory genes with the release of chemokines [7, 8]. The importance of another damage mechanism, apoptosis, in the pathogenesis of pulmonary contusion has been better understood in recent years [9]. It is known that alveolar epithelial apoptosis increases after lung contusion and there is a decrease in alveolar type 2 cells due to apoptosis in the lungs of animals 48 h after blunt chest trauma [10, 11].

In trauma-induced contusion, tissue inflammation and increased intracellular oxidative stress are observed. Oxidative stress and mitochondrial damage mutually exacerbate each other, contributing to further cellular dysfunction in the contused tissue. The mitochondria, responsible for energy production, become compromised, further aggravating cellular injury and impairing overall energy metabolism, which can contribute to the progression of tissue damage [12–14].

In tissues with high energy metabolism such as lung tissue, mitochondrial stress-induced apoptosis often develops in response to damage. During increased oxidative stress and inflammation, the Bax/Bcl-2 ratio increases. This changes leads to increased mitochondrial membrane permeability and the release of cytochrome-c into the cytoplasm, ultimately activating caspase-9 (Cas-9). Caspases are enzymes that sequentially activate one another along the apoptotic pathway. Like other caspases, Cas-9 triggers the activation of caspase-3 (Cas-3), which is the terminal caspase responsible for the execution of apoptosis [15, 16]. Consequently, oxidative stress and inflammation—key drivers of apoptosis, particularly in the context of thoracic trauma—were prioritized in this study, given their critical role and the potential benefits of mitigating cell death to enhance the healing process.

**Fig. 1:**
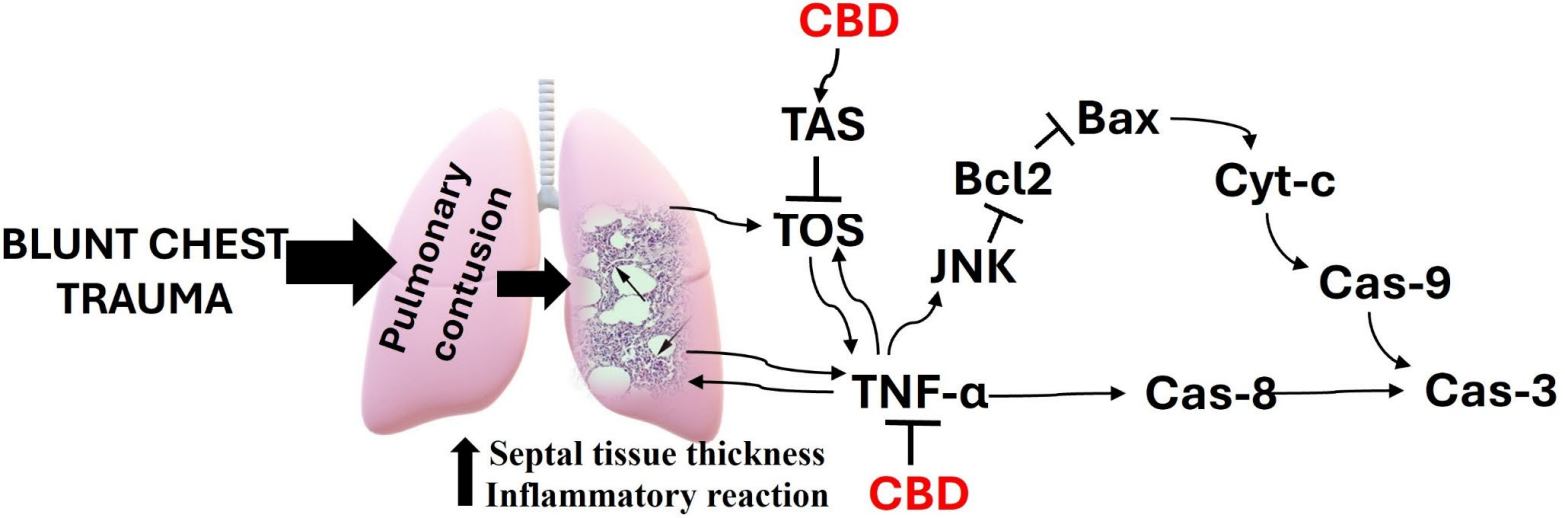
The mechanism of CBD protection against blunt chest trauma-induced acute lung injury. CBD: Cannabidiol, TOS: Total oxidant status, TAS: Total antioxidant status, TNF-α: Tumor necrosis factor-alpha, JNK: c-Jun N-terminal kinase, Bcl-2: B-cell lymphoma 2, Bax: Bcl-2 associated X protein, Cyt-c: Cytochrome-c, Cas-9: Caspase 9, Cas-3: Caspase 3, Cas-8: Caspase 8

Cannabidiol (CBD) is an important nonpsychotropic cannabinoid component of cannabis (*Cannabis sativa*). The discovery of cannabinoid receptors led to the characterization of the endocannabinoid system which modulates a variety of physiological activities, such as immunomodulation and inflammation reduction. CBD may exert its effects through various intracellular pathways in addition to inflammation and oxidative stress redution [17–20]. The action mechanism of damage and how CBD effects, is demostrated in Fig. 1.

Given its high mitochondrial activity, we focused on the Bax/Bcl-2/Cas-9 pathway, which mediates apoptosis through mitochondrial stress. This study aimed to investigate the hypothesis that CBD, with its known anti-inflammatory and anti-apoptosis effects, reduces the severity of ALI in patients with pulmonary contusion following blunt chest trauma.

## Materials and methods

### Ethical approval

The protocols for animal research were followed in all of the animal studies we conducted for this study: Reporting in Vivo Experiments (ARRIVE) 2.0 at all stages of the experiment. The Suleyman Demirel University local animal experimentation ethics committee approved the experimental methodology (number: 26.01.2023/116-01) and the experiments were carried out in accordance with this protocol. Suleyman Demirel University’s Scientific Research Projects Coordination Unit provided funding with project code TSG-2023-9010.

### Reagents

The CBD was delivered from Suleyman Demirel University, Natural Products Application and Research Center. The source of the CBD was the extract of *Cannabis sativa L.* (Cannabaceae). The CBD content was > 99.9, and the tetrahydrocannabinol content was < 0.01. The limits of residual alcohol and heavy metals comply with USP and EU pharmacopeias. To induce sedation and anesthesia, Xylazin Bio %2 (Bioveta, Czech Republic), and Keta-Control (Doğa Ilac, Turkey) were used.

### Animals and experimental design

Forty adult Wistar albino male rats weighing 350–400 g used in the experiments were housed at 21–22 °C and %60 ± %5 humidity with a 12-hour light:12-hour dark cycle and were fed with standard commercial feed and water ad libitum. All rats were divided randomly into 4 groups (each containing ten rats) after they were obtained from Suleyman Demirel University Experimental Animals Laboratory. All rats were anesthetized with 50 mg/kg ketamine and 10 mg/kg xylazine before the model application. CBD doses were also selected from the previous studies [21, 22]. Groups as follows:

1-Sham group: 0.1 ml solvent solution (%0.9 NaCl + Tween 80) was administered intraperitoneally (i.p.). 30 min later, anesthesia was administered; however, no trauma was induced.

2-Trauma group: 0.1 ml of solvent solution was administered i.p., and 30 min later, pulmonary trauma was induced under anesthesia.

3-Trauma + CBD group: 5 mg/kg CBD in a 0.1 ml volume of solvent solution was applied i.p. and 30 min later, pulmonary trauma was induced under anesthesia.

4-CBD group: 5 mg/kg CBD in a 0.1 ml volume of solvent solution was applied i.p. and 30 min later, anesthesia was administered; however, no trauma was induced.

For anesthesia during sacrification, ip 80 mg/kg ketamine (Keta-Control, Doğa İlaç, Turkey) and 10 mg/kg xylazine (Xylazin Bio 2%, Bioveta, Czech Republic) were applied. After 48 h the trauma, rats were sacrificed under anesthesia. Following the abdominal incision, euthanasia was performed by surgical exsanguination with blood taken from the vena cava inferior and right lung tissues were removed. Later, half of the removed lung tissues were preserved in %10 buffered formalin under conditions suitable for histopathological and immunohistochemical analysis. The remaining lung tissues were stored at -20 °C for biochemical analyses and at -80 °C for genetic analyses.

### Blunt chest trauma model creation

**Fig. 2:**
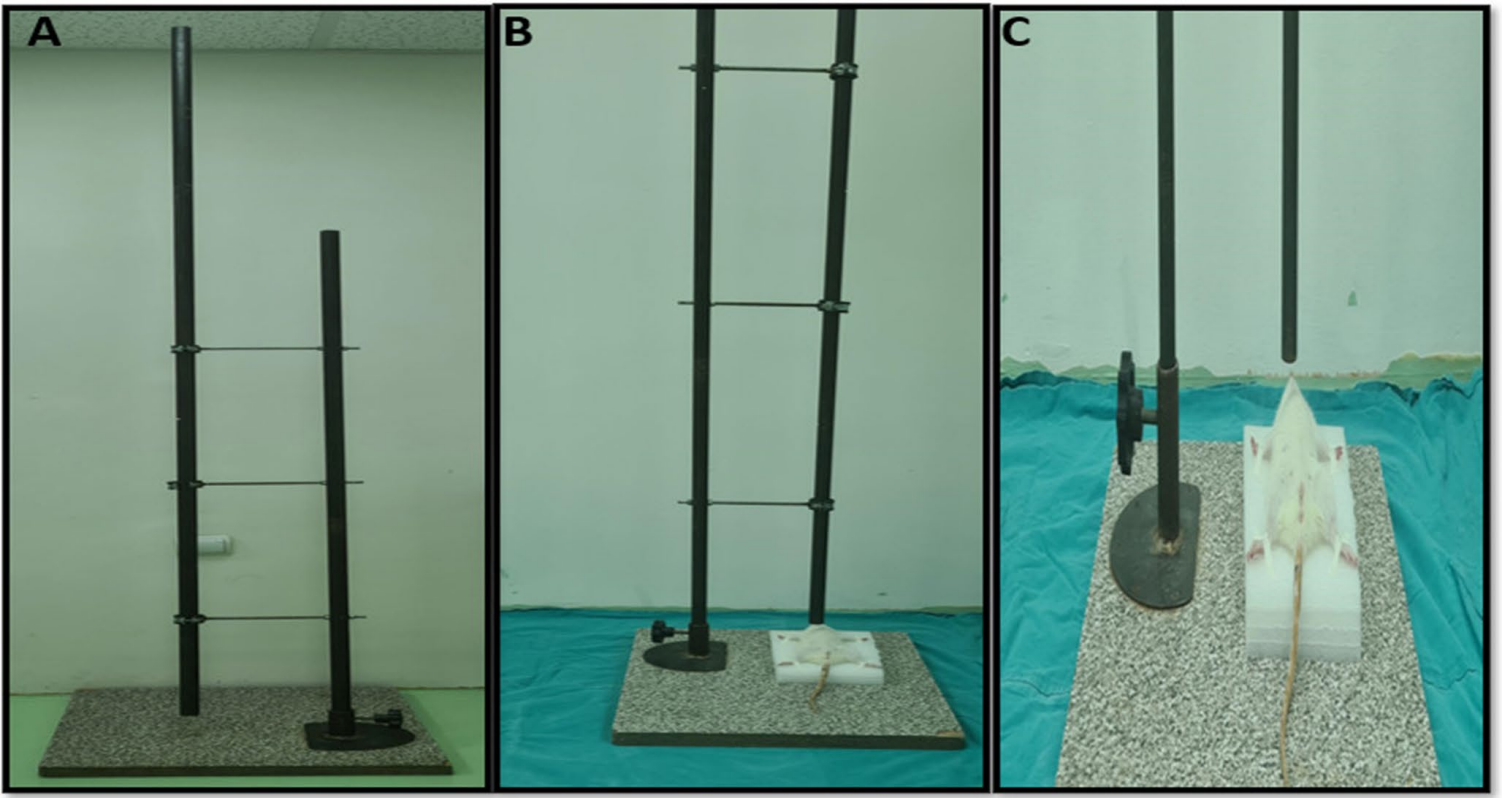
Application of the blunt chest trauma model in rats. (**A**) Experimental setup, (**B**, **C**) Placing rats in the experimental setup and positioning them

The blunt chest trauma model was implemented as previously described in other studies [23]. Briefly, a 200 g weight from a height of 1 m was dropped onto the right anterior thoracic wall. The resulting energy was calculated via the formula E = mgh (where E represents energy, g is the gravitational acceleration at 9.8 m/s², h is the height at 100 cm, and m is the drop weight at 0.2 kg). With a weight of 200 g and a height of 1 m, the energy transferred to the chest wall was calculated as 1.96 joules. Thus, pulmonary trauma has been induced in rats by applying 1.96 joules of energy (Fig. 2).

### Histopathological analyses

All lung tissue samples were taken routine histological tissue processing procedure using a fully automated tissue processing device (Leica ASP300S; Leica Microsystem, Nussloch, Germany), and then all samples were embedded in paraffin wax. After chilling the paraffin blocks 5 μm sections taken using a rotary microtome (Leica RM 2155; Leica Microsystem, Nussloch, Germany) from paraffin blocks. Hematoxylin-Eosin (HE) staining and a coverslip were applied, followed by examination under a light microscope. The histopathological lesions were scored on a scale of 0 to 3 based on their severity of hyperemia, edema, inflammatory cell infiltration, and epithelial cell loss.

### Immunohistochemical examination

Furthermore, two sets of sections from every paraffin block were cut and taken on slides coated with poly-L-lysine and immunohistochemically stained to detect the expression of caspase-3 (Recombinant Anti-Caspase-3 antibody [EPR18297] (ab184787)) and TNF-α (Recombinant Anti-TNF alpha antibody [RM1005] (ab307164)) using the streptavidin-biotin method as directed by the manufacturer. Each primary antibody diluted to 1/100. Sections were incubated with the primary antibodies overnight. Then, streptavidin-alkaline phosphatase conjugate and a biotinylated secondary antibody were used for immunohistochemistry. As a secondary antibody, we used the Mouse and Rabbit Specific HRP/AEC (ABC) Detection IHC Kit (ab93705). The chromogen employed was aminoethyl carbazole (AEC). The source of all primary and secondary antibodies was Abcam, Abcam (Cambridge, UK). Instead of using primary antibodies for negative controls, an antigen dilution solution was applied. A trained pathologist from another university conducted each test on blinded samples.

At an objective magnification of X40, the percentage of cells that were positively immunostained for each marker in 10 different areas for each slide for all groups was computed. Utilizing the ImageJ software (National Institutes of Health, Bethesda, MD, version 1.48), counting was performed on the image analyzer’s output. Before counting, the images were cropped, divided into color channels, and any artifacts removed. After being chosen using a selection tool, cells inside the regions of interest were counted using the software’s counting tool. The red color was used to identify positive staining, and only cells with strong red staining were considered positive. Microphotographs were taken using the Database Manual Cell Sens Life Science Imaging Software System (Olympus Co., Tokyo, Japan).

### Biochemical analyzes

Lung tissue specimens were processed by homogenization employing the Ultra Turrax Janke & Kunkel homogenizer (IKA^®^ Werke, Germany) for oxidant-antioxidant analysis.

Utilizing commercial kits obtained from Rel Assay Diagnostics (Gaziantep, Turkey), spectrophotometric measurements of Total Antioxidant Status (TAS) and Total Oxidant Status (TOS) were performed using the Beckman Coulter AU 5800 autoanalyzer (Beckman Coulter, USA). Calculation of the Oxidative Stress Index (OSI) was accomplished utilizing the formula OSI= [(TOS/TAS)×100] [24]. TAS and TOS analysis have been performed according to Erel’s protocols [25, 26]. The outcomes were quantified in units per gram of protein.

### RT qPCR analyzes

Total RNA was obtained using the RNA isolation kit (Nepenthe, Turkey). The purity and quality of RNAs were measured by nanodrop (Shimadzu Ltd. Kyoto, Japan). cDNA was synthesized using 1 µg of RNA (Atlas Biotechnology, Turkey). Specific mRNA primer sequences were determined using the NCBI website (Table 1). Gene expression levels were determined using a real-time PCR instrument (Biorad CFX Connect, California, USA) with 2X SYBR green master mix (Nepenthe, Turkey). The manufacturer’s instructions were followed in the preparation of the reaction mixture. For normalization, the GAPDH gene was used as housekeeping. Relative mRNA levels were calculated using the 2-ΔΔCt [27].

**Table 1:** Primary sequences, product size and accession numbers of genes

Genes	Primary sequence	product size	accession number
GAPDH (HouseKeeping)	F: AGTGCCAGCCTCGTCTCATA	248 bp	NM_017008.4
	R: GATGGTGATGGGTTTCCCGT		
Bcl-2	F: GGTGAACTGGGGGAGGATTG	102 bp	NM_016993.2
	R: AGAGCGATGTTGTCCACCAG		
Bax	F: TTGCTACAGGGTTTCATCCA	112 bp	NM_017059.2
	R: GACACTCGCTCAGCTTCTTG		
Cas-9	F: AGCCAGATGCTGTCCCATAC	151 bp	XM_017597018.2
	R: CAGGAACCGCTCTTCTTGTC		

F: Forward, R: Reverse, GAPDH: glyceraldehyde-3-phosphate dehydrogenase, Bcl-2: B-cell lymphoma 2, Bax: Bcl-2 associated X protein, Cas-9: Caspase 9

### Statistical analyses

For statistical analysis, the one-way ANOVA with post-hoc LSD tests was used by the Graphpad Prism 8 (San Diego, California, USA). A significance threshold of *p* < 0.05 was accepted.

## Results

### Macroscopic examination

**Fig. 3:**
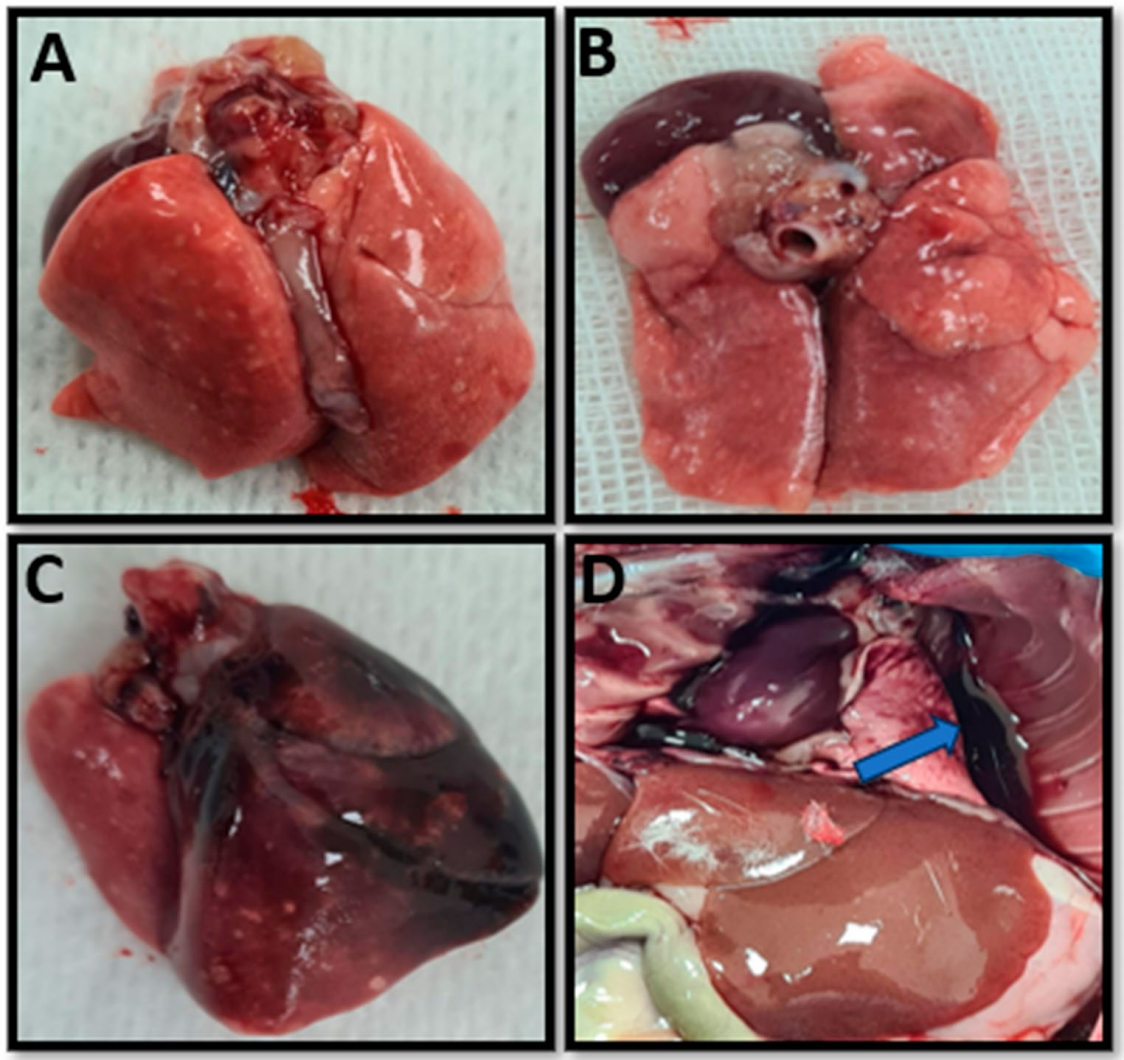
Macroscopic view of lung tissues. (**A**) Anterior view of the lungs of a rat from the Trauma + CBD group (**B**) Posterior view of the lungs of a rat from the Trauma + CBD group (**C**) Anterior view of the lungs of a rat from the Trauma group (**D**) Post-sternotomy view of a rat from the Trauma group (blue arrow shows hemothorax)

After sternotomy, the lungs of the rats were evaluated macroscopically. In the lung tissues of the Trauma group, hemorrhagic foci were widespread and the lungs of 5 (%50) rats were dark red, 3 (%30) rats were bright red, and 2 (%20) rats were dark pink. At the same time, hemothorax was observed in 3 (%30) rats in the Trauma group. In the Trauma + CBD group, only 2 (%20) hemothoraxes were observed, and the lungs of 6 (60%) rats in this group were light pink, 2 (%20) were dark pink and the remaining 2 (%20) were bright red (Fig. 3).

### Histopathological and immunohistochemical results

**Fig. 4:**
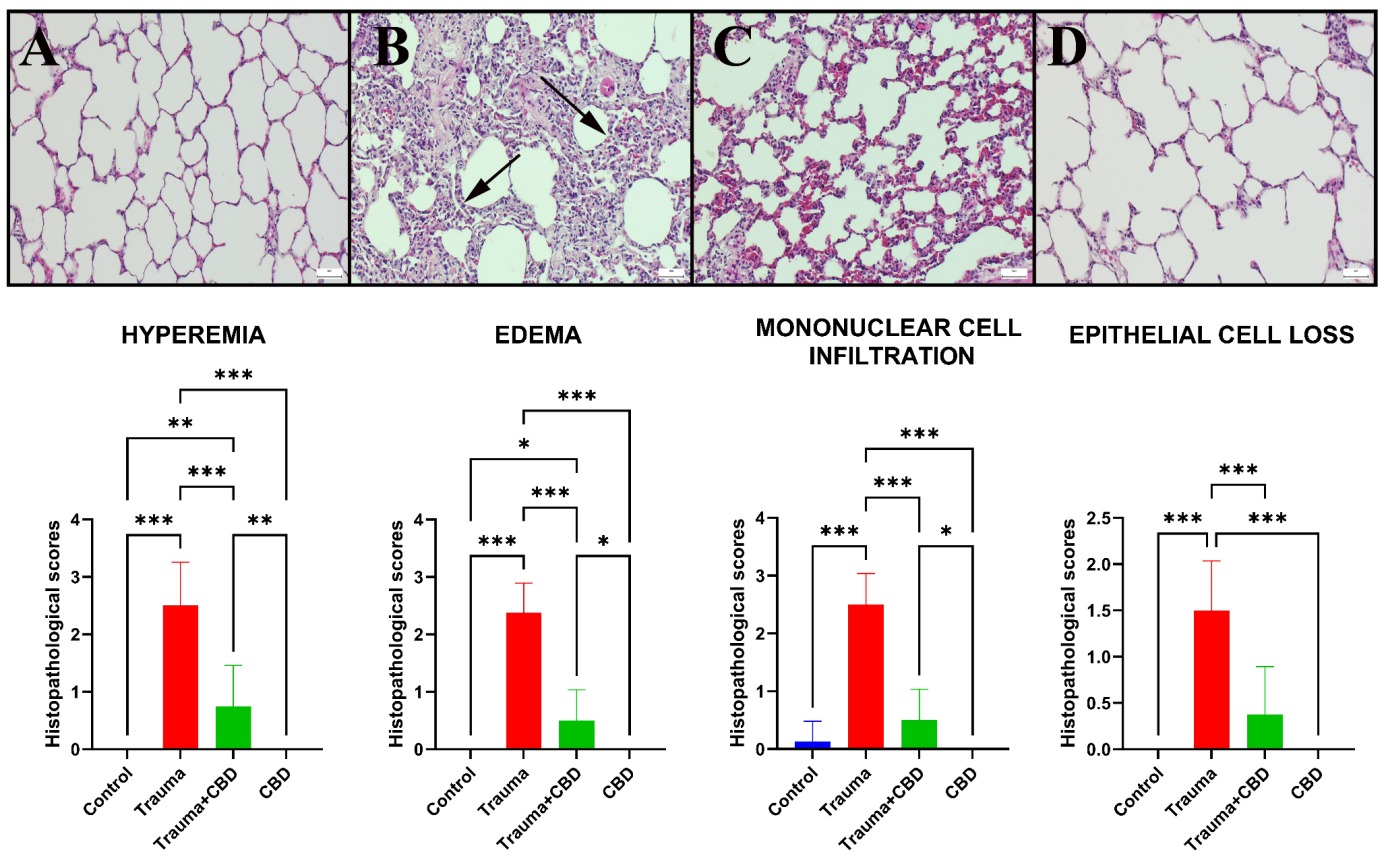
Histopathological view of lungs among the groups. (**A**) Normal lung histology in the Sham group. (**B**) Increased septal tissue thickness and inflammatory reactions (arrows) in the lungs of the Trauma group. (**C**) Decreased inflammatory reaction and septal tissue thickness in the Trauma + CBD group, (**D**) Normal tissue architecture in the CBD group, HE, scale bars = 50 μm. Statistical analysis of immunohistochemical values performed with One-way ANOVA (post-hoc Tukey). Values are presented as means ± SD. *p < 0.05 **p < 0.01 ***p < 0.001, Error bars represent standard deviation

Histopathological examination of lung sections from the sham and CBD groups revealed normal tissue microarchitecture. The Trauma group displayed marked emphysema, pronounced hyperemia, markedly increased septal tissue thickness, and severe inflammatory cell infiltration, which was composed mainly of neutrophil leukocytes compared to sham group (*p* < 0.001 for all). CBD medication in the Trauma-CBD group lessened the harmful outcomes (*p* < 0.001 for all) (Fig. 4).

**Fig. 5:**
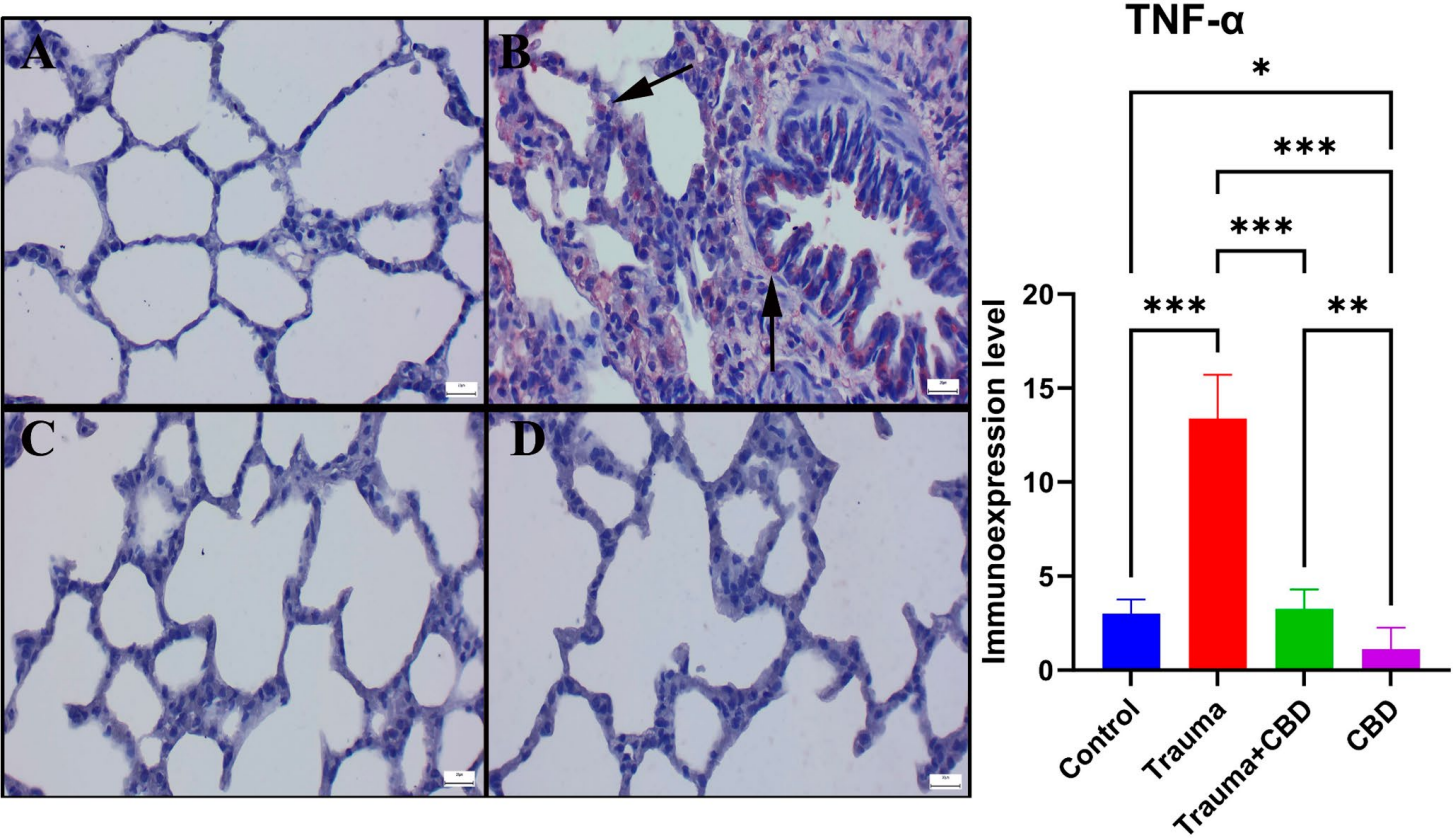
Comparison of TNF-α immunoexpression in the lungs between the groups. (**A**) Negative expression in the Sham group. (**B**) A marked increase in expression (arrows) of TNF-α staining was detected in the Trauma group. (**C**) Decreased expression (arrow) in the Trauma + CBD group. (**D**) No immunoreaction occurred in the CBD group. Streptavidin-biotin peroxidase method; scale bars = 20 μm. Statistical analysis of immunohistochemical values performed with One-way ANOVA (post-hoc Tukey). Values are presented as means ± SD. TNF-α: Tumor necrosis factor alpha, *p < 0.05 **p < 0.01 ***p < 0.001, Error bars represent standard deviation

**Fig. 6:**
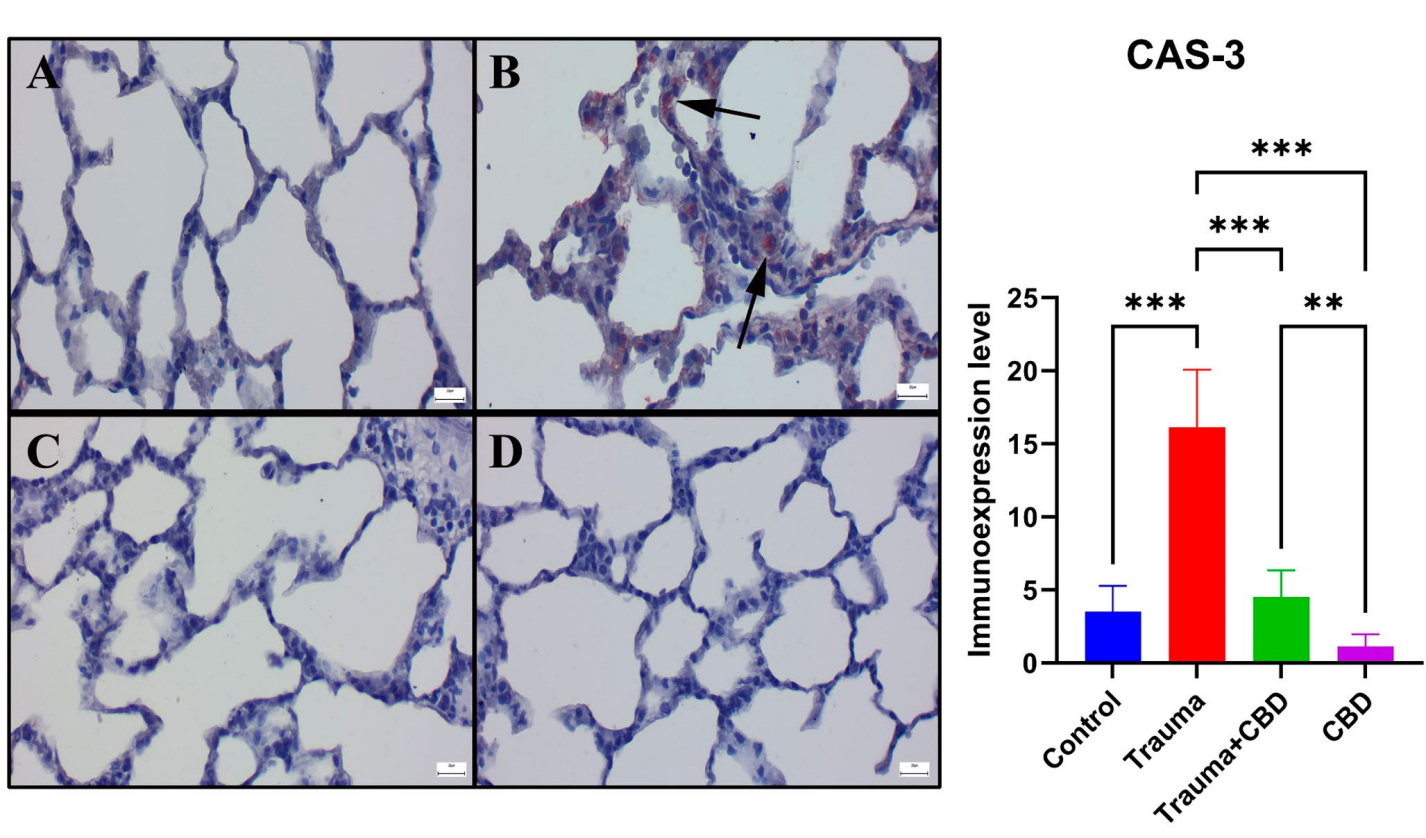
Comparison of caspase-3 immunoexpression in the lungs between the groups. (**A**) Negative expression in the Sham group. (**B**) A marked increase in expression (arrows) of Cas-3 staining was dedected the Trauma group. (**C**) Decreased expression (arrow) in the Trauma + CBD group. (**D**) No immunoreaction occurred in the CBD group. Streptavidin-biotin peroxidase method; scale bars = 20 μm. Statistical analysis of immunohistochemical values performed with One-way ANOVA (post-hoc Tukey). Values are presented as means ± SD. Cas-3: Caspase 3, *p < 0.05 **p < 0.01 ***p < 0.001, Error bars represent standard deviation

When the Sham group’s immunohistochemically stained slides were examined, there was no or very little Cas-3 and TNF-α expression were observed. The Cas-3 and TNF-α immunoexpressions were significantly elevated in the Trauma group compared to sham group (*p* < 0.001 for both). Additionally, following CBD treatment, the expression of Cas-3 and TNF-α significantly decreased in Trauma + CBD group compared to Trauma group (*p* < 0.001 for both). The CBD group showed similar expressions to the Sham group by means of markers (Figs. 5 and 6). Alveolar epithelial cells, alveolar macrophages, and inflammatory cells were the common sites of expression.

### Biochemical results

**Fig. 7:**
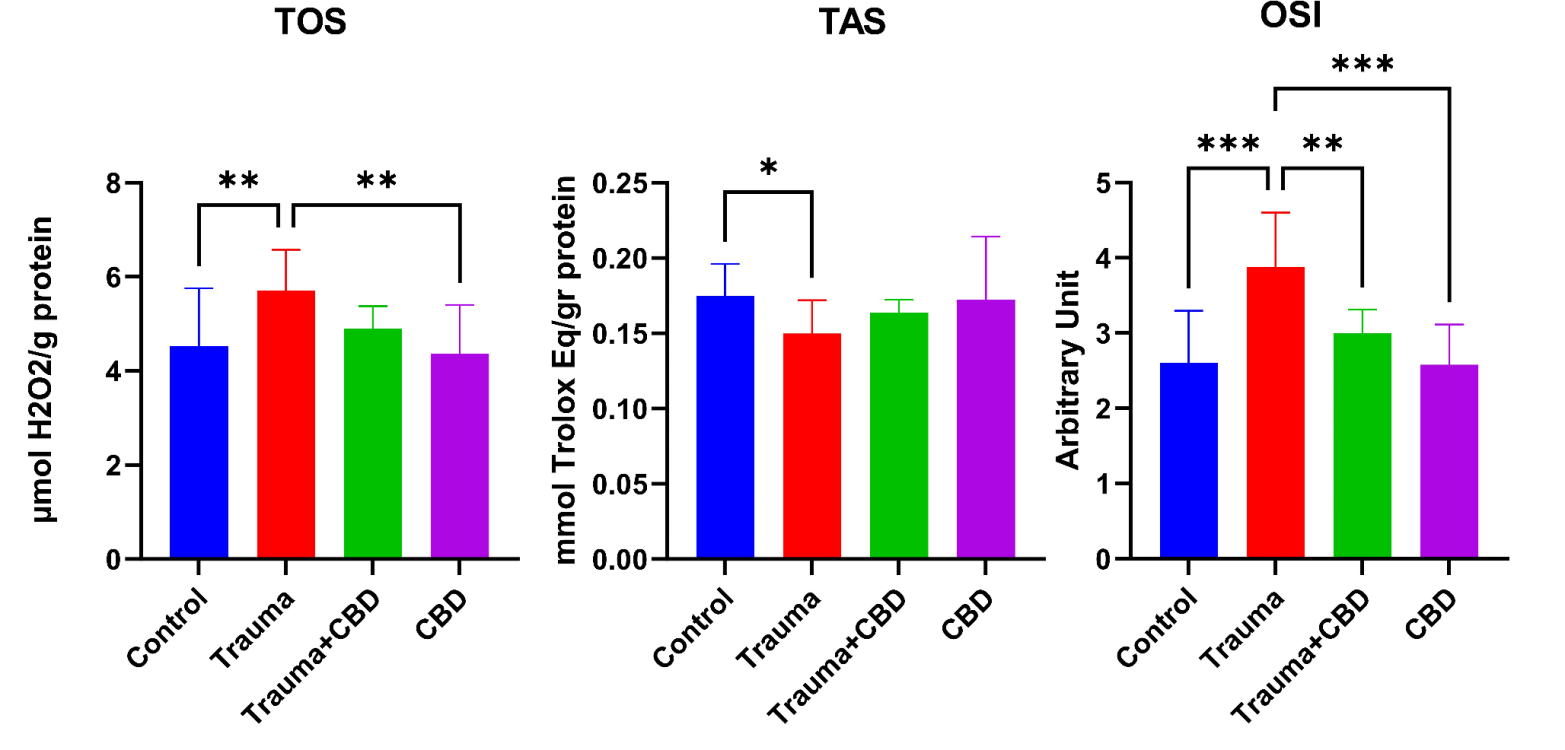
Oxidative stress parameters of lung tssues. The values are presented as the means ± SD. The relationships between groups and the results of biochemical marker analyses were assessed by one-way ANOVA and post hoc LSD tests. CBD: Cannabidiol, TOS: Total oxidant status, TAS: Total antioxidant status, OSI: Oxidative stress index. “*” represents *p* < 0.05, “**” represents *p* < 0.01, and “***” represents *p* < 0.001

In the Trauma group of this study, TOS and OSI levels were increased and TAS levels were decreased compared to the sham group significantly (*p* = 0.008, *p* < 0.001, and *p* = 0.042; respectively). In addition, TOS and OSI levels were significantly higher in the Trauma group compared to the CBD group (*p* = 0.003 and *p* < 0.001; respectively). In the Trauma + CBD group, OSI levels were significantly lower compared to the Trauma group (*p* = 0.002) (Fig. 7).

### RT qPCR results

**Fig. 8:**
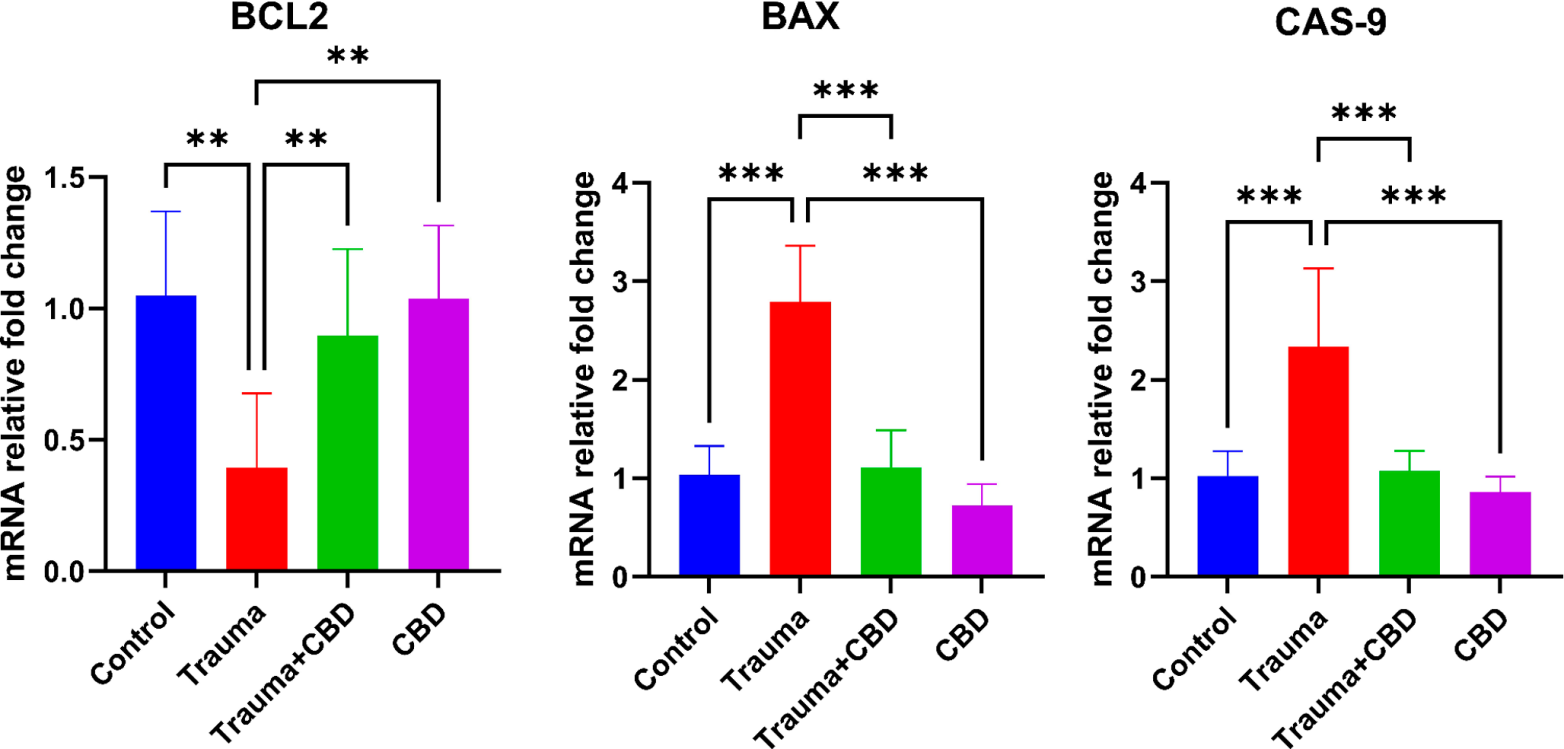
mRNA relative fold change graph of genes. The values are presented as the means ± SD. Statistical analysis was performed with one-way ANOVA and post hoc LSD test. Bcl-2: B-cell lymphoma 2, Bax: Bcl-2 associated X protein, Cas-9: Caspase 9, CBD: Cannabidiol. “**” represents *p* < 0.01, “***” represents *p* < 0.001

In the Trauma group, expressions of Bcl-2 were significantly decreased compared to the Sham, Trauma + CBD, and CBD groups (*p* = 0.001, *p* = 0.009, *p* = 0.002; respectively). Conversely, expressions of Bax were significantly increased in the Trauma group compared to the other three groups (*p* < 0.001 for all groups). Similar to Bax expressions, Cas-9 expressions in the Trauma group were significantly increased compared to the Sham, Trauma + CBD, and CBD groups (*p* < 0.001 for all) (Fig. 8).

## Discussion

Various pathologies, such as alveolar distension, alveolar rupture, separation of alveoli from bronchioles, intra-alveolar hemorrhage, and interstitial edema caused by chest trauma are called pulmonary contusion [5]. Secondary lung tissue damage after trauma-induced injury develops with attenuated inflammation and apoptosis [7].

Raghavendran et al. reported an increased number of leukocytes (predominantly neutrophils) in the alveolar spaces and interstitium 24 h after the contusion caused by blunt chest trauma in their animal experiment, with prominent neutrophilic infiltration observed at 48 h and septal thickening in the alveoli [28]. In another model of ALI induced by lipopolysaccharide in mice diffuse morphologic damage, such as hemorrhage in lung tissues, interstitial edema, thickening of the alveolar septal tissue, and infiltration of polymorphic leukocytes into the parenchyma and alveolar spaces were reported [29]. In this study, it was observed that emphysema, hyperemia, and septal tissue thickness decreased in the CBD treated group, and inflammatory cell infiltration was at a lower level compared to the Trauma group. Macroscopically, these findings were confirmed by observing that hyperemia decreased and the tissue preserved its vitality.

In the acute phase of pulmonary contusion, the migration of many inflammatory cells, especially neutrophils, is observed [28]. Infiltrating neutrophils may increase necrosis/apoptosis of cells in the alveolar epithelium via different mechanisms and thus lead to oxidant-mediated damage and disruption of alveolar fluid transport function [7]. In ALI, neutrophils accumulate excessively in the alveoli and pose a great difficulty in resolving the inflammation [9]. The Trauma + CBD group showed a substantial reduction in inflammatory cell migration as compared to the Trauma group. Thus, CBD was largely beneficial in preventing inflammatory cell-mediated damage in contusion. Considering the inflammatory responses mentioned above, CBD may reduce the inflammatory response in acute traumatic lung injury, and these positive effects are supported by macroscopic images.

In addition to histopathological findings, TNF-α, which is immunohistochemically stained, is a pleiotropic cytokine that plays a role in various lung pathologies including fibrosis, emphysema, asthma, and smoking-related connective tissue damage [30]. Neighboring cells that secrete TNF-α are stimulated to create more powerful proinflammatory chemokines and cytokines, including IL-6, macrophage inflammatory proteins, cytokine-induced neutrophils, keratinocyte-derived chemokines, and monocyte chemotactic proteins [29]. Xing et al. reported an increase in TNF-α levels in lung tissue in lung injury induced by lipopolysaccharide in mice [31]. In the Trauma group, there was a marked increase in TNF-α levels. At the same time, the fact that TNF-α levels decreased in the CBD treated group revealed that CBD showed its anti-inflammatory effect in this way. Concurrently, this circumstance is corroborated by the CBD group’s reduced TNF-α levels in comparison to the sham group. When the TNF-α results are evaluated alongside histopathological findings, it is evident that CBD has significant effects on inflammation in the affected tissue.

One of the first organelles in cells affected by inflammation and impaired oxygenation is the mitochondrion, which is responsible for cellular energy metabolism. Oxygen acts as the final electron acceptor; therefore, mitochondrial dysfunction often results in the production of reactive oxygen species (ROS) along with other free radicals. These ROS and free radicals, formed both in the cytoplasm and within the mitochondria, attack essential cellular structures such as DNA, enzymes, and the membranes of the cell and organelles within. This disruption in cellular integrity and function is referred to as oxidative stress [32].

Free radicals generated during inflammation, necroptosis, and apoptosis at the contusion site affect oxidant levels, antioxidant enzyme activities, and the balance between these two parameters, i.e. the OSI [6]. In this model, OSI values showed a significant increase in the Trauma group than in the other groups. OSI values were significantly reduced in the Trauma + CBD group compared to the Trauma group. However, no significant difference was observed in the TAS and TOS values between the Trauma + CBD group compared to the Trauma group. These indicators suggest that CBD alone has little antioxidant effect on the lung, but that has antioxidant activity in the presence of inflammation. On the other hand, it can be said that the antioxidant effects may be increased by increasing the dose of CBD used in this study, which is an acute injury model, or by repeated use. Similar to our study Bauer et al., in their study including in vitro and in vivo experiments, showed that CBD reduced oxidative stress and early apoptosis in the lung due to thoracic irradiation [33].

Oxidative stress can also occur in normally functioning cells; however, the damage is mitigated by intracellular antioxidant enzyme systems. When the extent of damage exceeds the cell’s antioxidant capacity, mitochondrial dysfunction progresses [32]. Another damage mechanism in secondary lung injury occurring after contusion is increased apoptosis [7]. Apoptosis can occur via both the extrinsic pathway and the internal mitochondrial pathway. In both pathways, cell death occurs with cas-3 activation. Caspases are a family of enzymes that activate one another in a sequential manner along the apoptotic pathway, ultimately leading to programmed cell death. Cas-9, like other caspases, initiates the activation of cas-3, the final executor of apoptosis. In the extrinsic pathway, caspase activation occurs with an external stimulus or damage to the cell, and in the intrinsic mitochondrial pathway, cas-3 activation is performed by cas-9 stimulated by cytochrome-c.

. The mitochondrial, intrinsic, apoptotic pathway is regulated by the Bcl-2 protein family, which includes pro-apoptotic members such as Bax and Bak, as well as anti-apoptotic members like Bcl-2. While Bcl-2 maintains membrane stabilization, Bax promotes increased permeability. When a death signal received in response to the described damage mechanisms, Bcl-2 levels decrease and causes Bax to form pores on the mitochondrial membrane. This process leads to the release of apoptotic molecules such as cytochrome-c into the cytosol, triggering caspase activation and ultimately resulting in apoptosis [15, 34].

Recent studies have shown that many aspects of mitochondrial biology, including mitochondrial dynamics, are critical determinants of the genesis and progression of lung disease [35]. In this model, we suggest that CBD may inhibit apoptosis specifically through the mitochondrial pathway. This was evidenced by the increase in cas-3 in the Trauma group and the decrease in the amount of cas-3 in the CBD treated group. At the same time, the greater increase in the amount of Bcl-2 and the significant decrease in the amount of Bax and cas-9 in the CBD treated group than in the Trauma group showed that CBD exerts this antiapoptotic effect, especially through inhibition of the mitochondrial pathway. We believe that the large amount of vascular structure in the lung tissue and the large number of mitochondria in the lung cells potentiate these effects. The mechanism by which CBD protects against blunt chest trauma-induced ALI is summarized in the graphical abstract.

Other studies have shown that CBD reduces apoptosis by modulating oxidative stress and inflammation across various pathologies, with applications ranging from acute to chronic use [33, 36–39]. Several of these studies have investigated distinct cellular pathways to uncover the underlying mechanisms. In a study by Ozmen et al., the effects of CBD on lung injury secondary to cardiac ischemia were investigated via the endoplasmic reticulum stress pathway [39]. Similarly, Hosseinzadeh et al. examined intrathecal CBD treatment in epileptic rats. Significant improvements in seizure activity were observed with repeated daily CBD administration, while its effects on autophagy-related proteins and antioxidant responses were evident from the very first dose [37]. In a study by Kang et al., it was reported that CBD prevents mitochondrial dysfunction by inducing autophagy [18]. We believe that CBD’s protective effects on these pathways are mostly driven by its capacity to mitigate oxidative stress and suppress inflammatory activity.

The limitation of this study is that we only touched on some of the pathways affected by CBD within the cell. The direct molecular interactions of CBD with intracellular and extracellular molecules were beyond the scope of this research and are left for future studies. Another limitation is how the effects of CBD vary depending on time and dose. CBD, which has shown therapeutic effects in many tissues in the literature, needs to be studied in other tissues and experimental models, and other intracellular pathways, along with application time and dose differences, need to be elucidated.

## Conclusion

In conclusion, it has been observed that CBD reduces lung damage in lung contusions caused by blunt chest trauma through its anti-inflammatory and antiapoptotic effects. In addition, the effects of a single dose of CBD were examined in this study, and more detailed molecular studies are needed in which longer-term use or higher doses are preferred, in addition to this study, which highlights the acute effects of CBD. The ability to perform analyses at the gene level at the protein level via the western blot method will increase the effectiveness of the study.

## Data Availability

The data of this research is available upon request to the Corresponding author [S.E.A].
